# Capturing temporal heterogeneity of communities: A temporal β-diversity based on Hill numbers and time series analysis

**DOI:** 10.1371/journal.pone.0292574

**Published:** 2025-08-12

**Authors:** Daniel J. Sánchez-Ochoa, Edgar J. González, María del Coro Arizmendi, Patricia Koleff, Raúl Martell-Dubois, Jorge A. Meave, Hibraim A. Pérez-Mendoza

**Affiliations:** 1 Laboratorio de Ecología Evolutiva y Conservación de Anfibios y Reptiles, Facultad de Estudios Superiores Iztacala, Universidad Autónoma de México, Los Reyes Iztacala, Tlalnepantla, Mexico; 2 Posgrado en Ciencias Biológicas, Unidad de Posgrado, Circuito de Posgrados, Coyoacán, Ciudad Universitaria, Mexico City, Mexico; 3 Departamento de Ecología y Recursos Naturales, Facultad de Ciencias, Universidad Nacional Autónoma de México, Coyoacán, Mexico City, Mexico; 4 Laboratorio de Ecología, UBIPRO, Facultad de Estudios Superiores Iztacala, Universidad Nacional Autónoma de México, Tlalnepantla de Baz, Mexico; 5 Comisión Nacional para el Conocimiento y Uso de la Biodiversidad, Tlalpan, Mexico City, Mexico; University of Santiago, CHILE

## Abstract

Beta-diversity is a term used to refer to the heterogeneity in the composition of species through space or time. Despite a consensus on the advantages of measuring β-diversity using data on species abundances through Hill numbers, we still lack a measure of temporal β-diversity based on this framework. In this paper, we present the mathematical basis for a temporal β-diversity measure, based on both signal processing and Hill numbers theory through the partition of temporal ƴ-diversity. The proposed measure was tested in four hypothetical simulated communities with species varying in temporal concurrence and abundance and two empirical data sets. The values of each simulation reflected community heterogeneity and changes in abundance over time. In terms of ƴ-diversity, *q*-values are closely related to total richness (S) and show a negative exponential pattern when they increase. For α-diversity, *q*-value profiles were more variable than ƴ-diversity, and different decaying patterns in α-diversity can be observed among simulations. Temporal β-diversity shows different patterns, which are principally related to the rate of change between ƴ- and α-diversity. Our framework provides a direct and objective approach for comparing the heterogeneity of temporal community patterns; this measure can be interpreted as the effective number of completely different unique communities over the sampling period indicating either a larger variety of community structures or higher species heterogeneity through time. This method can be applied to any ecological community that has been monitored over time.

## Introduction

We live in a biodiverse world where species changes along space and time [1,2]. The different forms of biodiversity have been studied using different mathematical, statistical and information system approaches [3,4]. The principal goal of all these measures is to characterize variation in biodiversity across different spatial and temporal scales [5–7]. The regional component of biodiversity (γ diversity) contains the inter-site differences or biodiversity heterogeneity (β diversity) between local richness of species (α diversity). These three components interconnected and together reflect how species are distributed across communities. Thus, the study of γ, α and β diversities provide insights that can predict changes in biodiversity [8–13]. These concepts have applications like understanding species dispersion, hotspot regionalization, reserve design, and clarification of the complementarity of the composition of species; they are thus considered ones of the most important concepts of ecological theory [2,3,13–19]. However, while γ and α diversity are conceptually similar, β diversity revolves around the heterogeneity of biodiversity patterns throughout different ways.

There are two main analytical approaches for measuring β-diversity. The first one is the decomposition approach, in which β-diversity is calculated based on the segregation of ƴ-diversity and α-diversity components (i.e., γ = α * β or γ = α + β) to understand how distinctly the communities are in terms of richness and evenness; this approach can be used to address questions related to the heterogeneity and the number of unique species in the communities in a landscape, or to the proportion of species that are not shared among all sampling units. The second one includes differentiation or variance measures derived from the total similarity of a pairwise community abundance matrix comparing specific composition of species throughout turnover-nestedness concept [20], addressing questions related with proportion of gains and losses of species among spatial or temporal subsets of communities [21–23]. Both approaches allow different hypotheses related to the processes driving species distribution and diversity patterns to be tested [3,13,20,24,25]. Although there is much discussion about what measure is optimal for a given objective or purpose, it has been suggested that both approaches could provide complementary insights that contribute to the advancement of community theory [26–30]. However, the temporal axis of β-diversity has been less explored than the spatial axis, and even some authors have pointed out the lack of reliable temporal frameworks and measures [3,28].

Temporal diversity approaches of γ and α diversity often adapt common spatial indices such as Simpson, Shannon and Jaccard [29–32]. A recent and robust method for temporal α diversity, based on Hill numbers, captures the effective number of equally abundant species and can incorporate species traits [33]. However, temporal β-diversity lacks a unified framework [28]. Engen proposed bivariate correlations between community assemblages [22], while Baselga´s β_TUR_ and β_NES_ measures remain widely used to their reliance for presence/absence data, which suits low sampling effort [13]. Legendre used Moran´s Eigenvector Map analysis (MEMs) to asses correlations among time points in community samples [34–36]. And finally, a temporal β-diversity index was later proposed to quantify changes in species composition between adjacent time points [35].

Temporal axis presents new conceptual and methodological challenges compared to spatial axis [28]. Species abundance in the communities change over time due to environmental, demographic and stochastic contexts, making the temporal data to be dynamic and most of the time non-stationary [36]. This make difficult to distinguish between the natural variation and the directional changes of species, and introduces challenges with temporal autocorrelation and uneven or discontinuous samples, limiting the reliability of standard diversity indices.

To overcome these challenges, it is necessary to account frameworks for the temporal structure of data. [37,38]. Wavelet transform analysis, allows to decompose abundance signals to predict gaps in the data, modeling a continuous variable [39] This allows to model a continuous abundance process rather than discrete, permitting to standardize the abundances per species [28].

In this context, integrating time series analysis with diversity theory may potentially provide a reliable way to develop a measure for temporal beta diversity. Hill numbers are a group of parametric measures used to measure diversity based on the modification of the *q ≥* 0 parameter, which determines the sensitivity of the measure to the relative abundance of species [20,40]. Because Hill numbers express the effective number of species richness-abundance distributions, they provide a unified and interpretable framework that can be extended beyond taxonomic diversity to incorporate functional, phylogenetic and even phenological diversity, depending on the similarities among traits [15,21,26,41,42].

A measure of temporal diversity based on Hill numbers and time series analysis that captures the heterogeneity of diversity through time could provide a more promising strategy for assessing this important biodiversity component, similar to the way in which spatial diversity measures have become comparable among studies [8,40]. We expect that these measures will enhance estimates of temporal γ and α diversity, and have a new temporal β that allows interpretable comparisons among studies, considering the ecological processes as a continuous variable. In addition, the measures could be used to characterize temporal diversity patterns and thus provide insights into the temporal dynamics of communities; information on temporal compositional shifts can shed on light on the status of communities and the effects of environmental variables on temporal species heterogeneity optimizing time and resources in monitoring and management plans [28,35,43]. Therefore, the objectives of this work is to (1) develop a temporal beta diversity metric based in time series and Hill numbers analysis, (2) to evaluate its performance through simulated datasets and (3) to test its applicability using empirical field data in order to explore the utility for describing and comparing temporal patterns of biodiversity across ecological communities.

## Methods

### Validity of the stationarity of the time series of species abundance

The verification of stationary is pivotal for the standardization of the species abundance time series data. Generally, ecological data exhibit variability in their stationarity characteristics, often resulting in a limited degree of comparability when working with time series. For instance, we performed a stationarity test analysis (Dickey-Fuller test) to know the nature of species abundance curves data using a confidence level of 0.05. We conducted a Dickey-Fuller stationarity analysis to each species abundance dataset to determine whether the temporal data need to be standardized through time series analysis (S1 File). If at least one time series was stationary, we proceeded with the next sections.

### Temporal patterns as a continuous variable: temporal diversity data preparation

We developed our temporal α, γ and β-diversity measures based on the wavelet transform analysis [17,44,45] and the Hill numbers diversity approach [26,40,46]. Our proposed temporal diversity measures were developed specifically for the decomposition approach. Assuming stationarity of species abundance data and the gaps associated with the discrete sampling effort, we used wavelet analysis to estimate a continuous abundance curve, enabling the use of distance-based Hill numbers [26], with the area under the curve as a pairwise distance metric.

### τ – the rate of change of species abundances over continuous time

**Fig 1:**
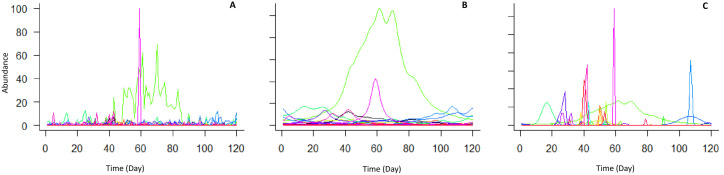
Demonstration of a same data set of abundance patterns of a Malagasy amphibian community. (A) representation of the raw data, (B) wavelet transformed data with ***τ*** = 2, and (C) wavelet transformed data with adjusted ***τ*** values. Each color represents a species (S = 40; N = 120 days).

Biological processes occur gradually and continuously, however in most cases they are recorded discretely due to sampling constrains. Nevertheless, there are differences in rates of change in the occurrence and abundance changes of species. For example, groups of species that are highly responsive to environmental changes such as amphibians, reptiles and insects, often show abrupt and rapid fluctuations in their abundances. In contrast, organisms like plants often show more attenuated patterns of abundance change. Being more specific, within these general patterns, each species exhibits particularities in the temporal dynamics. Thus, ***τ*** is introduced as a species –specific parameter that captures this biological variation. (Fig 1, S1 File).

Wavelet analysis typically assigns a value of 2 to the rates of change weighting in modeled phenomenon through time. This default value arises from the properties of the link wavelet function, where a balance between time and frequency resolution is optimized with a value of 2 (valuable for physicists and engineers). However, since species differ in the steepness and speed of their abundance fluctuations due to their natural history traits, assigning a fixed value is not always biologically appropriate. Instead, a unique ***τ*** should be assigned to each species to better account to this heterogeneity.

Wavelet analysis allows interpolation and smoothing of a continuously modeled abundance curve. However, unlike conventional wavelet analysis applications, our objective requires explicit specification of the attenuation threshold (***τ***), a parameter that controls for the rate of continuous change in the occurrence of species in the community. In other words, ***τ*** determines the slope (i.e., the speed) at which species abundance or species intensity changes through time [47,48].

For instance, some species whithin a community may display faster rates of appearance compared to others [49], and thus a uniform ***τ*** –value would obscure meaningful biological differences [50]. Therefore, instead of assuming a fixed parameter value (typically 2 in most analyses), a species-specific ***τ*** value is needed [45,51,52].

The ***τ*** value must consider the sampling resolution (time interval between observations), the total sampling duration (T), and the observed steepness of the abundance changes. Thus, if time series data have high temporal resolution, ***τ*** will be more sensitive to species-specific rate of change; conversely, in low resolution datasets, ***τ***-values will depend more heavily on the smoothed abundance curve estimated via wavelet transform analysis.

Here, we propose, for a given *i* species, the calculation


$$\tau_{i}=\left(\tau_{max}-\tau_{min}\right)\delta_{i}+\tau_{min},$$ eqn. 1

where


$$\tau_{max}={\left(T-1\right)}^{\frac{T}{T+1}},$$ eqn. 2

$\tau_{\max}$ corresponds to a constant of the maximum value that $\tau_{i}$ can take in the analyzed community, $\tau_{min}=2$, $\tau_{min}$ corresponds to the constant minimum threshold that $\tau_{i}$ can take and T represents the total number of sampling times.

The equation


$$\delta_{i}=d_{i}-\left(d_{i}\right)/\left(d_{i}\right)-\left(d_{i}\right),$$ eqn. 3

represents the rescaling factor between 0–1 of $d_{i}$, where min_i_ and max_i_ are the minimum and max values of each $d_{i}$; or the derivative that biologically refers to the rate of change where


$$d_{i}=\frac{1}{T}\int_{1\to t}^{T}\int \left|\frac{d^{2}z_{i}(t)}{dt^{2}}\right|dt,$$ eqn. 4

$d_{i}$ corresponds to the mean concavity of absolute values of the species abundance; in other words, $d_{i}$ is the average steepness of the changes in species abundances between consecutive samples in time. The integer has the minimum value of zero and the maximum T. Finally, $z_{i}(t)$ represents the simulated abundance smoothed curve of the *i-*th species for each time, whereas *T* represents the total number of sampling times.

Each $\tau_{i}$ value combines the species-specific abundance change rate and the maximum number of sampling points of the study, thus balancing between resolution and observed data. The scaling function used for the wavelet analysis was the Morlet scaling function, which is optimal for data with unknown frequencies and scales, and data that cannot be directly interpreted [48].

### Temporal β diversity: effective number of distinct communities over time

For our temporal β-diversity measure, we replaced the discrete abundance vectors by abundance or intensity curves derived from the wavelet time series analysis. We modified the equations from Chao & Chiu ([21]; eqn. 5), so that the relative abundance values (*z*_*i*_) represent the abundance curves of the **i*-th* species. To estimate temporal β-diversity through the multiplicative component (ƴ/α), we first need to calculate temporal ƴ-diversity and temporal α-diversity. To calculate ƴ-diversity of order *q* (^*q*^*DTγ*), we used the relative abundance of species in the community ($z_{i+}$*/*$z_{++}$; *i* = 1, 2, …, *S*), where $z_{i+}=\int_{T}z_{i}(t)dt$ is the total area of abundance of *i-th* species, and $z_{++}=\sum_{i=1}^{S}\int_{T}z_{i}(t)dt$ is the total sum of the area abundance of all species (*S*). Consequently, ƴ-diversity of order *q* is defined as


$$\begin{array}{c} qDT_{\gamma T}={\left\{\sum {\mathrm{\backslash nolimits}}_{i=1}^{S}{\left(\frac{z_{i+}}{z_{++}}\right)}^{q}\right\}}^{\frac{1}{\left(1-q\right)}}, \end{array}$$ eqn. 5

and when *q* = 1 as


$$\begin{array}{c} 1{DT}_{\gamma T}=exp\left\{-\sum {\mathrm{\backslash nolimits}}_{i=1}^{S}\frac{z_{i+}}{z_{++}}loglog\left(\frac{z_{i+}}{z_{++}}\right)\right\}, \end{array}$$ eqn. 6

The temporal ƴ-diversity is interpreted as the “effective number of species in the entire community through time” or the species richness when q = 0. For temporal α-diversity we applied the same set of measures and definitions proposed by Chao & Chiu [21] but on a temporal scale. In this sense, temporal α-diversity represents “the effective number of species per time unit” or the “mean effective number of species per time unit”, and defined by:


$$q{DT}_{\alpha T}=\frac{1}{T}{\left\{\sum_{i=1}^{S}\int_{T}{\left(\frac{z_{i}(t)}{z_{++}}\right)}^{q}dt\right\}}^{\frac{1}{\left(1-q\right)}},$$ eqn. 7

and when *q* = 1, as:


$$1{DT}_{\alpha T}=exp\left\{-\sum_{i=1}^{S}\int_{T}\left(\frac{z_{i}(t)}{z_{++}}\right)log\left(\frac{z_{i}(t)}{z_{++}}\right)dt-log(T)\right\}.$$ eqn. 8

Finally, the multiplicative temporal β-diversity can be calculated as:


$$qD_{\beta_{T}}=q{DT}_{\gamma T}/q{DT}_{\alpha T},$$ eqn. 9

This value can be interpreted as “effective number of completely different unique communities over the sampling period”. The contribution of species heterogeneity among communities is based on changes in the rate of whole community richness and the mean community changes at each sampling point. Temporal α- and ƴ-diversities always range from > 0 to *S* and decrease as *q* increases; temporal β-diversity ranges from 1 (when *q* = 0) to infinite.

To illustrate the use and utility of this measure of temporal β-diversity, we performed simulations designed to challenge the model under contrasting conditions. We generated scenarios that pushed the limits of the method by varying the abundance and heterogeneity of species richness over time (we include in data files in S2 File).

In addition, we performed two extra analyses based on field data of an amphibian community from Madagascar (*S* = 40; time period = 360 days; frequency = daily; [53] and a macro-benthic community from Chesapeake Bay (*S* = 66; **time period* *= 24 years, frequency = yearly; Chesapeake Bay Foundation) [54].

We used R Studio with the DescTools [55] and wavScalogram [56] packages. The script for the temporal β-diversity calculation can be found in S3 R File.

## Results

Patterns of temporal α- and ƴ-diversity in the simulations were consistent to those suggested by other diversity measures based on Hill numbers. The values of each simulation reflected community heterogeneity and changes in abundance over time. In terms of ƴ-diversity, *q*-values were closely related to total richness (S) and showed a negative exponential pattern when they increase, except when species abundances remained constant over time.

**Fig 2:**
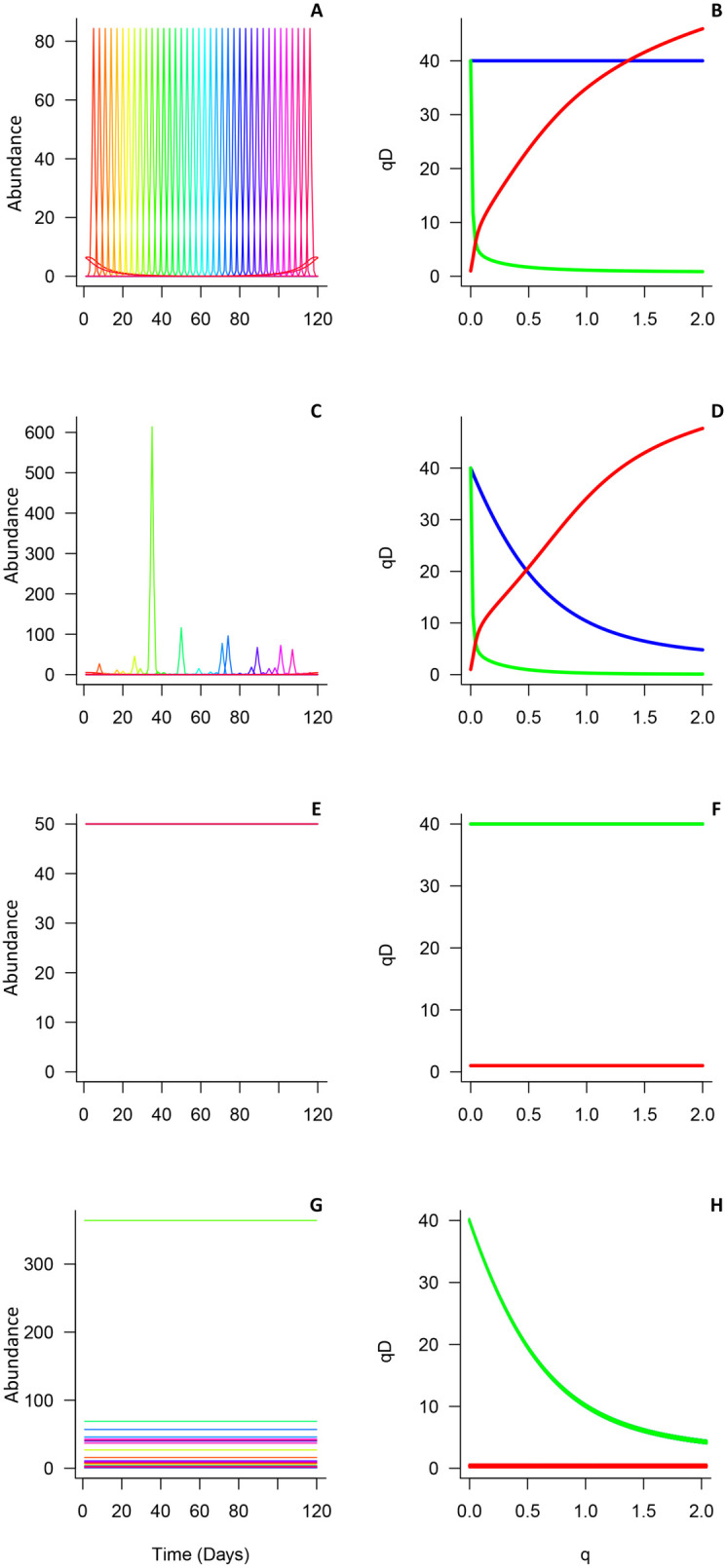
Simulations tested in temporal beta diversity. Graphs showing simulated species-abundance distributions over time (left panels) and the corresponding temporal ƴ-, α-, and β-diversity profiles as functions of q, for 0 ≤ **q* *≤ 2 (right panels): In the top panels: (A, B) species are equally distributed over time and abundance patterns are identical, representing a situation where each species can be found at any specific time; second row panels (C, D) species are equally distributed over time and abundances are unequal; the third row (E,F) species are present all the time without abundance variation and equal abundances; and bottom panels (G, H) species are present all the time without abundance variation and unequal abundances. In left panels (B, D, F and H: Temporal ƴ- (blue line), α- (green line) and β-diversity (red line).

For α-diversity, *q*-value profiles were more variable than for ƴ-diversity. Different decaying patterns in α-diversity were observed among simulations (Fig 2). When species abundance varied, α-diversity consistently decay while q is increasing (Fig 2B, 2D and 2H). In contrast, the absence of decreasing trend Fig 2 reflects the null variation in species richness or abundance over time.

The minimum β-diversity value was always 1 and generally increased as *q* increases, except in simulations where neither ƴ- nor α- diversity changed (i.e., Fig 2F and 2H). The most variable β-diversity pattern appeared in Fig 2B and 2D, corresponding to simulations with highly heterogeneous temporal changes in species abundances, where species were evenly redistributed across time.

**Fig 3:**
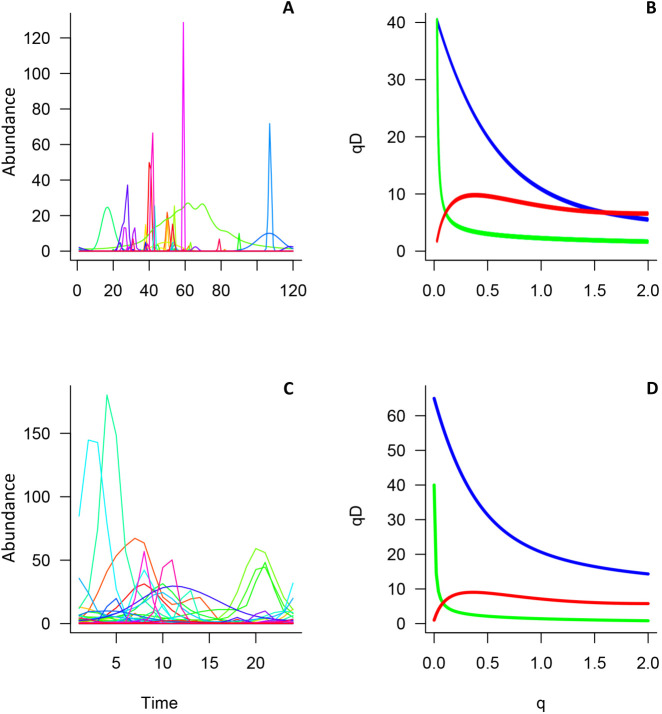
Graphs showing the real data sets of species abundance distributions over time. The left panels corresponds to temporal ƴ-, α- and β-diversity profiles as functions of q, for0 ≤ **q* *≤ 2 (right panels). In the top panels **(A)** Malagasy amphibian community (S = 40); bottom panels **(B)** Chesapeake macro benthos community (S = 66). In left panels (B, D: Temporal ƴ- (blue line), α- (green line) and β-diversity (red line) profiles.

We found that four species exhibited stationarity abundance time series. Detailed results of the stationary tests are presented in S2. For the Malagasy amphibian community, temporal β-diversity shows high values near *q* = 0 and then stabilizes as *q* increases; by contrast, β-diversity shows a nearly constant increasing pattern as *q* increases in the Chesapeake Bay macro-benthic community (Fig 3).

## Discussion

Here, we propose a temporal γ-, α- and β-diversity measures based on time series analysis and Hill numbers diversity theory, providing an interpretable, efficient and comparable framework to characterize community heterogeneity over time. To improve robustness and address issues related to sampling effort [39,45], we apply wavelet analysis, which models gradual changes in species abundance across multiple temporal scales. A major challenge in temporal diversity analysis, especially β-diversity, is that datasets often fail to meet time series assumptions as they overlook sampling resolution, time frequency characteristics, and stationarity [35,57,58]. Previous studies have shown the usefulness of time series approaches for analyzing ecological and forestry data [51,59] including population dynamics, disease transmission, animal migration and phenology [44,47,51,60–63]. Given the greatly distinct patterns in species temporal activity, a method that can summarize such heterogeneity is essential. We achieved this by approximating discrete abundance time series to continuous abundance curves, following approaches used in previous studies [37,47,51,64], and applying to the Hill numbers theory.

We achieved this by approximating discrete abundance time series to continuous abundance curves, following approaches used in previous studies [37,47,51,64] and applying to the Hill numbers. However, two key assumptions must be carefully evaluated when applying wavelet transforms: (1) equidistant temporal sampling and (2) sufficiently long time series data. While a minimum of 25 temporal points is recommended, we successfully applied the method to a dataset with 24 points (Chesapeake microbenthic community) [37,65]. However, there is no systematic evaluation of this assumptions, and future studies should address this using high resolution datasets by selectively removing sampling points. Although wavelet analysis is effective under these conditions, alternative methods such as Hilbert-Huang transform may be more appropriate for unevenly sampled data [66].

In our study, *τ*-values near 2 imply that the analyzed species have abundance patterns closely aligned with those modeled by wavelet analysis, and species with higher *τ*-values have simulated abundance curves that resemble the mode (statistics) of the raw data. This implies that species with more erratic o low detection patterns over time tend to have lower ***τ*** values, where wavelet transform plays a greater role in shaping the abundance curve. Thus, ***τ*** reflects not only the smoothing of biological process but also the degree of temporal coherence in species abundances patterns (β diversity calculation), which may be influenced by detection limitations or data sparsity.

This is particularly relevant for given the diversity in species activity patterns and detectability. Imperfect detection affects the accuracy of abundances estimates, as species may be present but remain undetected, leading to β diversity underestimates [67–69]. This has long been recognized in ecological monitoring [37,47,70]. In our analysis, the modeled curves tend to assign low values during time intervals where no detections occurred, thereby reducing false positives. However, these curves are still based on observed data, and no not explicitly incorporate detectability. Accounting for species time detection probabilities over time could refine our temporal β estimates, adding a new but necessary layer of complexity.

Temporal α diversity refers to the mean effective number of species observed per sampling unit. This approach based on Chao [26], indirectly accounts for detectability by reflecting the average number of species detected across time points, making it more sensitive to temporal variation in richness and species abundance. When q = 0 α and γ diversity values converge because the wavelet-based abundance curves assign near-zero probabilities to undetected time intervals, thus capturing imperfect detectability. This assumes species can be detected at any time, with variation shaped by species traits, spatial and temporal heterogeneity and sampling characteristics [71].

Although imperfect detection has been widely discussed in population and community studies [49,69,72,73]; the incorporation into biodiversity metrics is still limited [73,74]. Standardizing diversity values while accounting for rare species or species with low detectability remains necessary. From our experience, changing link function, ***τ*** parameter, or applying a cutoff value may refine estimates, but the overall diversity patterns remain consistent.

We built our framework based on a measure proposed by Chiu et al. [26], which was specifically designed to bridge the decomposition and differentiation perspectives of β-diversity. While our approach aligns with the decomposition framework, the mathematical formulation proposed by Chiu together with our time series perspective used in our study could be extended to develop a complementary differentiation-based perspective, adding new conceptual interpretation [15,75–77]. Likewise, other temporal β diversity frameworks, such as those of Baselga and Legendre [13,35], have demonstrated the value of using multiple measures of β diversity, but the utility of using multiple measures ultimately depends on the hypothesis tested [9]. However, the interpretation of these other measures requires caution because some refer to the concepts of turnover and others to variation (as our measure), but the interpretability is maintained under the Hill numbers framework. Otherwise, the most used measures of β diversity and even α diversity (Shannon and Simpson) [78] lack a structure that facilitates interpretation and comparison, [12,25].

In general, ƴ- and α- *q* profiles are consistent across estimated spatial diversity patterns [41]; however, *q*-profiles related to β-diversity do not show a consistent pattern. Specifically, temporal α-diversity only reflects an expected outcome rather than the reality indicated by the sampling measurements; thus, a completeness analysis could improve the robustness of the results for both temporal ƴ- and α-diversity as has been shown in other studies of diversity patterns [77,79,80]. For temporal β-diversity, higher values than S were observed in simulations, especially in cases where several species were equally distributed when *q* = 2. A high temporal β-diversity indicates a high number of unique communities throughout the sampling period and thus temporal heterogeneity in the activity of species within the community. Nevertheless, our measure is not suitable for indicating the moments where unique communities are occurring, but other measures can be used to provide this information, such as Legendre’s TBI (Temporal Beta Index) [35]. Thus, we show here that the use of different frameworks provides complementary information and that the use of each measure is not mutually exclusive.

Irrespectively to taxonomic or functional group, species richness, or temporal resolution, our approach to temporal β-diversity can be applied to abundance data. Although we expected asymptotic behaviors, we observed contrasting temporal β diversity patterns in the two data sets examined. In the Malagasy amphibian community, the temporal β-diversity *q*-profile shows high values when *q* is between 0 and 1, and the profile shows an asymptotic pattern. We initially expected that the differences between α- and ƴ-diversity would be relatively constant across q values, assuming that ƴ-diversity behaves similarly to α-diversity but a broader scale, and that the rate of change between them would remain stable. However, this was not the case. This supports previous findings by Mendes et al. [81], who showed from a spatial and α-diversity perspective that there are values of q (named q*) at which the diversity profiles changes more abruptly. In our case, this is reflected in sharper divergences between α and γ at specific q values. The rate of change in the temporal α-diversity q profile largely determines the heterogeneity of the community. In our example, few species of amphibians are commonly observed per sampling occasion or per unit of time; in other words, few species are recorded during each sampling event, and the species richness and abundance continuously vary as previously documented [53,82,83].

This result has direct implications for our understanding community heterogeneity through time, as well as for conservation and monitoring, since some community traits through spatial and temporal scales exhibit divergent patterns [84,85]. From a temporal perspective, we suspect a similar relation as in space: the difference between temporal γ and α defines temporal β diversity. So, if γ and α values are close each other β diversity remains relatively constant and low (close numerator and denominator values). In contrast, low α diversity (mean number of species per time) per unit time (distant numerator and denominator values) implies high β indicating a temporally heterogeneous community [3,86].

Communities that show high temporal heterogeneity in composition require conservation and monitoring plans which sampling frequency to capture the range of environmental variation.However, the reason for the need for a high sampling frequency is not solely because of temporal variation in the composition of communities but also because of variation in the detectability of species as aforementioned [87]. Low temporal α-diversity values do not indicate absence; rather, it is likely that features of the environment affect their conspicuousness [67,82,83] or even undergo short-distance migrations [88–90].

These dynamics are also tied to ecological processes such as interactions and phenological patterns. The occurrence of some species depends directly on the presence of other species [68]; as in the synchrony between flowering plants and pollinators [91,92], or predators prey [93–95] or parasites-host cycles [96]. Thus, comparing the temporal β diversity of different functional groups can test whether one group predicts the temporal β diversity of another. Our metric enables such comparisons and the formulation of new hypotheses.

Finally, in the case of the Chesapeake Bay macro-benthic community, we observed that the *q*-profile of the temporal β-diversity increases without reaching an asymptote; thus, temporal β-diversity values are likely higher than the one presented (8.17) and a higher sampling resolution or a longer time window could alter these results; ultimately, it is likely that this community is more heterogeneous through time. This demonstrates the need for more studies that estimate temporal β diversity using different levels of sampling effort or conducting analyses at different time scales to understand the effect of scale on temporal β diversity patterns, as other studies have shown that scale affects diversity patterns in other ecological axes [97–99].

## Conclusions

Our temporal diversity framework produces intuitive, comparable and simple values for assessing species heterogeneity over time. Our measure has the same properties of other γ, α and β-diversity measures and can be applied to mid- and long-term community data sets available for any taxon even on disturbed ecosystems. Understanding of temporal α-, β- and ƴ-diversities have important implications for the temporal design of community monitoring, conservation and restoration programs, even generating new questions related to temporal community changes and how communities are affected by this poorly explored axis. It would also be interesting to know whether temporal β-diversity responds similarly to spatial measures of temporal α- and γ-diversities. Exploring these could reveal general principles that govern diversity across both space and time. Also, future studies could test the inflection points observed in other q diversity profiles (e.g., Mendez with q*). Finally, the temporal diversity measure proposed here is suitable for analyzing communities across taxonomic level and temporal scale as long the sampling resolution and wavelet-related limitations are considered. Long species abundance time series would be particularly useful to compare diversity across years, seasons, or other cyclical intervals.

## Supporting information

S1 File Dickey-Fuller test and respective *τ* values of each species. (XLSX)

S2 File Simulations and study case databases. (XLSX)

S3 File Temporal β diversity script. qDBT is a measure of temporal beta diversity based on the framework of Chao & Chiu (2016). This tool calculates a descomposition temporal beta diversity measure from the Hill numbers perspective. First, the script transforms data on discrete species abundance patterns into modeled continuous patterns through wavelet analysis. Second, temporal gamma (qDG), alfa (qDA) and beta (qDB) diversity is calculated for these modelled species abundance patterns, where the “q” parameter controls the weight species intensity has on the diversity measure. (TXT)
